# Just When We Thought That COVID Was Over: A Systematic Review

**DOI:** 10.7759/cureus.27441

**Published:** 2022-07-29

**Authors:** Maha Tariq, Maitri V Acharekar, Sara E Guerrero Saldivia, Sumedha Unnikrishnan, Yeny Y Chavarria, Adebisi O Akindele, Ana P Jalkh, Aziza K Eastmond, Chaitra Shetty, Syed Muhammad Hannan A Rizvi, Joudi Sharaf, Kerry-Ann D Williams, Prachi Balani

**Affiliations:** 1 Research, California Institute of Behavioral Neurosciences & Psychology, Fairfield, USA

**Keywords:** physical medicine and rehabilitation, muscle aches, chronic fatigue, primary headaches, covid 19

## Abstract

As the globe continues to grapple and scuffle with new emerging strains of COVID every day, a set of recovered patients continue to show persistent enervating symptoms. Many patients never fully recovered after COVID and had neurological and psychiatric symptoms for weeks or months. The emphasis of our study is on these long haulers, particularly on the two critical organ systems of the body, i.e., the central nervous system and the muscular system. Depending upon the severity of the disease, many signs and symptoms continue to linger, ranging from weeks to months.

A total of 29 studies are included in our review after thorough screening, application of inclusion and exclusion criteria, and quality appraisals. The total number of patients included is 6012.

We found many long-term effects, but the emphasis of our study continued to remain on the two main organ systems that resulted in prolonged COVID with debilitating symptoms and thus affected the quality of life of these patients. Various factors and underlying pathophysiologic manifestations result in the predominance of these signs and symptoms.

Furthermore, the patient's underlying medical conditions and other environmental factors may add to it. More focus is required on the quality of life post-COVID, and this requires a team of specialists. There are still many unanswered questions like which ethnicity is affected more, why females are more prone to the long symptoms, and the effects of various treatments on the long-term signs and symptoms.

## Introduction and background

The severe acute respiratory syndrome coronavirus 2 (SARS-CoV-2) is a novel RNA β- coronavirus that causes COVID-19, started in late 2019, affecting millions of lives and causing a huge burden on the economy and physical wellbeing [1,2]. The virus, which was formerly known to affect the lungs and cause respiratory illness, in actuality affects multiple organs of the human body, causing various signs and symptoms [2,3]. A staggering number of patients experienced long-standing symptoms even after they were tested negative for the disease; due to this fact, it is referred to as long-COVID [1]. 

The most common symptoms experienced include; fatigue, headache, cognitive impairments, weakness, myalgia, poor concentration, and sleep disturbances [3-6]. The most likely mechanism through which this virus enters the brain is the angiotensin-converting enzyme receptor, thus causing profuse cytokine syndrome resulting in damage to the cells [4]. Apart from cytokine syndrome, autoantibodies against various body cells and autonomic nervous system disruption lead to post-viral illness [4,6].

The purpose of our study is to focus on the two main long-standing symptoms, fatigue and headaches, resulting in poor work performance and difficulty in performing daily activities of life.

Methods

**Figure 1:**
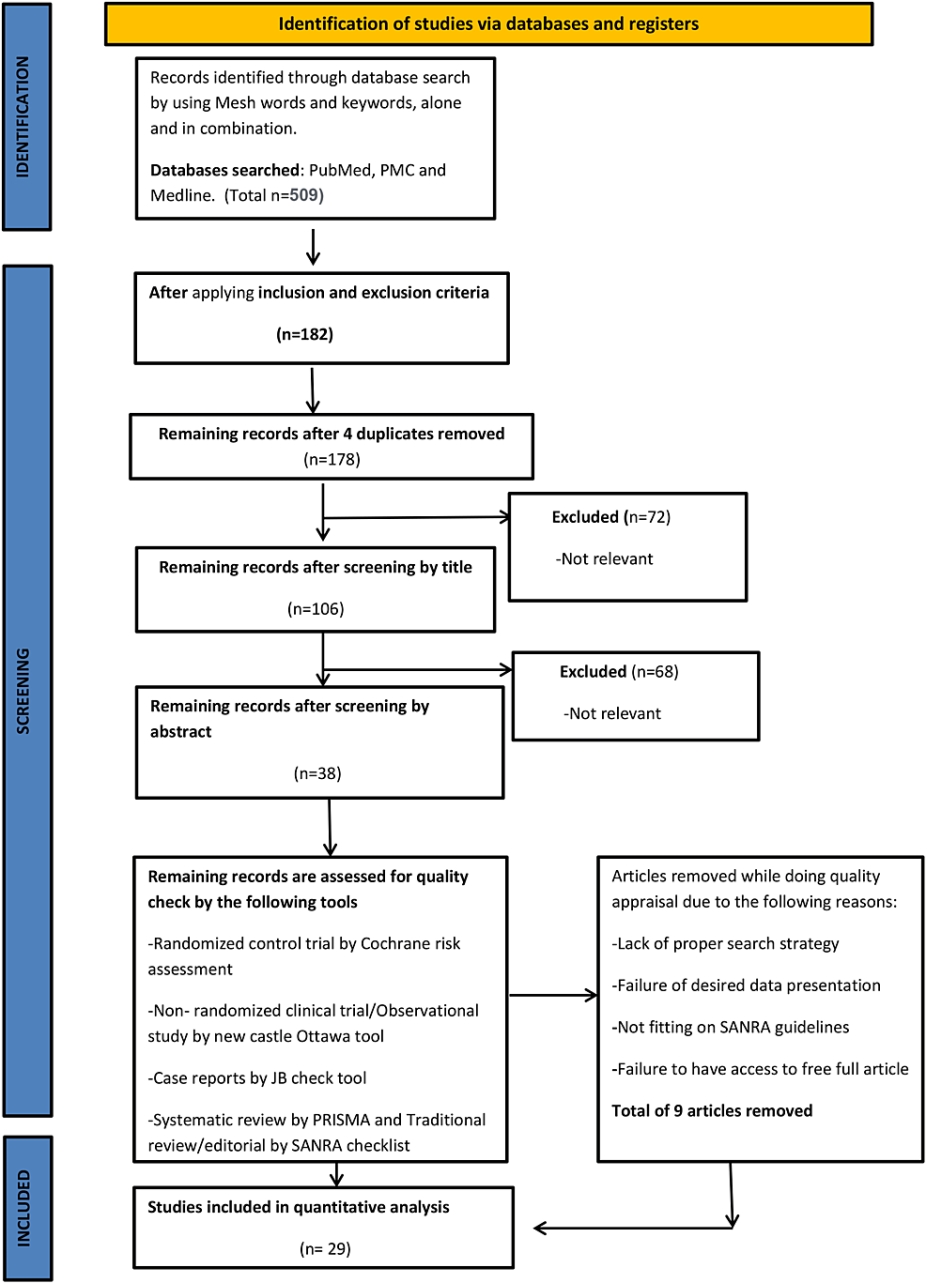
PRISMA flow chart PRISMA: Preferred Reporting Items for Systematic Review and Meta Analysis, MeSH: Medical Subject Heading, SANRA: scale for quality assessment of narrative review articles, PMC: PubMed Central

We obeyed the Preferred Reporting Items for Systematic reviews and Meta-Analysis (PRISMA) guidelines for conducting our systematic review (Figure 1) [7].

We systematically searched multiple electronic databases, such as PubMed, PubMed Central (PMC), and Medline, for data collection. We explored the databases by using terms of medical topics and by Medical Subject Headings (MeSH) words. The keywords used are “migraine,” “new-onset headache,” “exacerbation of headache,” “chronic fatigue syndrome,” “post-viral syndrome,” “lack of energy,” “exhaustion,” “fatigue,” “COVID19”, ”COVID recovered patients” and ”long term complications,” separately and in combination to find relevant studies. The total number of articles found in electronic databases is 509.

Inclusion and exclusion criteria

For our research, we included all the articles in English. Full free texts from the last three years were included. Studies that we included are clinical trials, randomized control trials, meta-analyses, and systematic and traditional reviews. Studies include humans only from age 18 and above. Studies done before 2019, gray literature, books, documents, and duplicates were excluded from the study.

Results

A total of 509 studies were obtained from the databases. Records were analyzed on the basis of title, abstract, and application of inclusion and exclusion criteria. A total of 182 studies were obtained. After the removal of duplicates, we were left with 178 studies. All the articles were further screened on the bases of title, abstract, and quality check; a total of 29 studies were left. The total number of patients included in the studies was 6012. Clinical trials were screened through the Cochrane risk bias assessment tool, observational studies by the Newcastle-Ottawa Scale, systematic and meta-analyses by PRISMA, and literature review by the scale for quality assessment of narrative review articles (SANRA) checklist.

## Review

The pathophysiology of coronavirus

Coronavirus is a single-stranded ribonucleic acid (RNA) virus with projections of glycoproteins on the outer surface. The main structure is composed of many proteins, with four proteins being the most important integral constituents, namely spike protein, M protein, nucleocapsid N glycoprotein, and E protein [4]. This virus gains entry into the human body through the nasal route. Once inside the body, it binds to the receptor named angiotensin-converting enzyme 2 (ACE2). As these receptors are located in the various cells of the body, this disease presents with a wide array of signs and symptoms [8].

This severe acute respiratory syndrome coronavirus 2 (SARS-CoV-2), or coronavirus disease (COVID-19), depending on its persistence of signs and symptoms, is classified as post-acute, with signs and symptoms ranging from about less than a month, and chronic, with signs and symptoms lasting for more than three months [1]. It has been seen that some patients who are recovered from COVID-19 still experience varying symptoms like fatigue, long-standing headaches, impaired cognition, generalized malaise, various psychiatric conditions, and many more. Therefore, this long-standing trail of symptoms is referred to as long COVID [5].

According to a study, T-cell dysfunction may be linked to the pathophysiology of long COVID, as seen in various autoimmune diseases. One of the mechanisms behind the pathophysiology of COVID-19 affecting other organs besides the lungs is the formation of antigen-antibody complexes, activating the humoral immune response [1].

**Figure 2:**
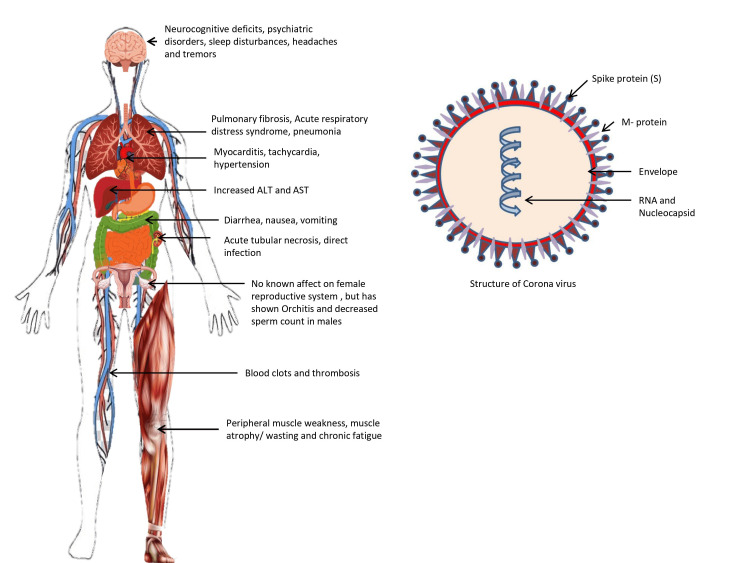
Structure of the virus and its effects on the body RNA: ribonucleic acid, ALT: alanine aminotransferase, AST: aspartate aminotransferase. Image created using Microsoft Powerpoint (Microsoft, Redmond, Washington)

Figure 2 shows the structure of the virus, how COVID-19 has affected various body organs, and what its various long-term residual symptoms are.

Effect on the central nervous system (CNS)

The virus gains entry into the brain through the olfactory bulb. The blood-brain-barrier (BBB) has ACE-2 receptors on the endothelial cells resulting in the viral entry into the nervous system, thus causing various signs and symptoms. The virus causes inflammation of the brain through a mechanism called cytokine storm syndrome.

Cytokine storm syndrome causes the percolation of various inflammatory cells resulting in increased amounts of cytokines, thus causing inflammation of the brain [1]. Apart from direct damage caused by the virus to the brain, tissue hypoxia and edema also leads to brain damage, as these findings were seen in the autopsy of patients deceased due to COVID-19 [1]. According to one of the studies, hypoxia leads to damage to the mitochondria, thus causing brain fog, i.e., altered cognition.

Neurological signs and symptoms of COVID-19 include headache, vomiting, brain fog, fatigue, behavioral problems, vertigo, loss of sense of taste and smell, and various psychiatric conditions; all of these conditions are referred to as neuro-COVID [1,8,9-12]. From all the studies which we have included in our review, headache is the most prevalent neurological complaint lasting from days to weeks post recovery. 

**Figure 3:**
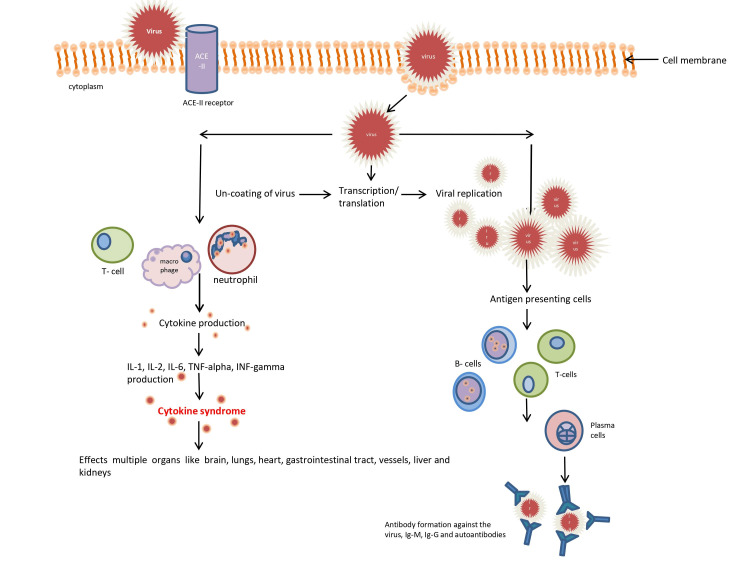
Entry of virus in the cell and chain of events following virus entry ACE-II: angiotensin-converting enzyme 2, IL: interleukin, TNF: tumor necrosis factor, INF: interferon, Ig: immunoglobulin Figure created using Microsoft Powerpoint (Microsoft, Redmond, Washington)

The chain of reactions occurring after the virus enters the body is shown in Figure 3

Effect on the peripheral muscles and mechanism of weakness and fatigue

One of the most important complications of COVID-19 is muscle weakness and unbearable fatigue. The presence of ACE-2 receptors on the skeletal muscles is the reason why skeletal muscles are affected by COVID-19 and thus present with continuing body aches. The muscle weakness could be due to the disease itself, whereas the sedentary mode of life due to the pandemic, long bed rest, and calorie deficit diet during the illness also contributed to the development of muscle weakness [8]. According to a study, brain fog, stringent fatigue, and other long-standing complaints of long COVID mirror myalgic encephalopathy or chronic fatigue syndrome, which develops after a viral infection [13]. Some studies have found autoantibodies against various cell lines and receptors aid in the austerity of symptoms. Along with that, involvement of the autonomic nervous system also pitches in the clinical picture of long COVID [10]. All this has led to poor quality of life. COVID-19 recovered patients cannot cope with daily activities and need help in carrying out their basic tasks. Rehabilitation is required for these patients so that they can return to their pre-COVID physical fitness level. Rehab includes a multifaceted approach which includes aerobic exercises, breathing exercises, proper sleep hygiene, and help with proper meal planning [9,10]. The use of multivitamins and as-needed analgesics for minor signs and symptoms of long COVID can also be helpful. 

**Figure 4:**
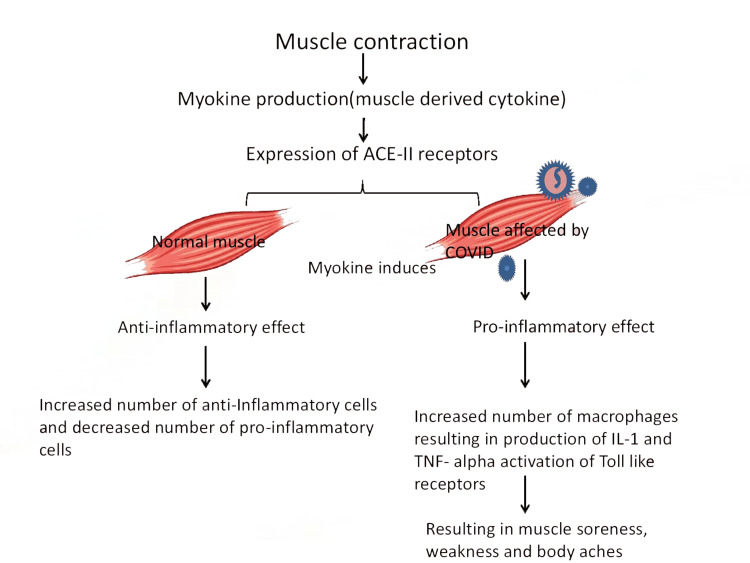
Muscle inflammation caused by the virus ACE-II: angiotensin-converting enzyme-2, IL: interleukin, TNF: tumor necrosis factor Image created using Microsoft power point (Microsoft, Redmond, Washington), [14]

Figure 4 explains how inflammation occurs in the muscle after being affected by the virus.

A list of all the studies included in the review is shown in Table 1.

**Table 1:** List of all the studies included in the review ACE: angiotensin-converting enzyme, ANS: autonomic nervous system, CNS: central nervous system, N/A: number of patients not mentioned in the study

Author and year	Type of study	Patients	Purpose of study	Results	Conclusion
Ahmad et al. [1], 2021	Systematic review	N/A	The long-term effects of COVID in recovered patients	COVID affects almost all the vital organs through antibody-mediated mechanism	A thorough clinical evaluation of COVID patients is required in order to fully understand its long-term effects
						Michelen et al. [2], 2021	Systematic review	N/A	The effects of long-standing COVID on different populations	Most common signs and symptoms of COVID include weakness, fatigue, impaired concentration, difficulty breathing, and abnormal lung function tests	Long COVID effects with wide variety of ongoing symptoms
												Becker et al. [3], 2021	Review	N/A	Autonomic nervous system dysregulation due to COVID	Resting increased heart rate, fatigue, and sleep disturbances could be due to disturbances in the autonomic nervous	Long-term fatigue and reduced exercise stamina could be a result of disruption of ANS
																		Nagu et al. [4], 2021	Review	N/A	Effect of COVID on CNS	Wide array of population had CNS involvement	COVID can lead to multiple effects on the CNS
Butler et al. [5], 2022	Review	N/A	Effects of COVID on the nervous system and cognition	Delirium is the most common neuropsychiatric complication of COVID	COVID has a great impact on cognition and nervous system function																		
																								Tirelli et al. [6], 2021	Clinical trial / multicenter study	100	Use of ozone therapy to treat post-COVID fatigue	Out of 100 patients, about 67 people benefited from the therapy	Ozone therapy can be used as a treatment agent
Korompoki et al. [8], 2021	Review	N/A	Long-term effects of COVID on different body organs	COVID has lead to a wide array of signs and symptoms	Almost all the organs of the body are affected by COVID																								
						Stefano et al. [9], 2021	Review/Historical article	N/A	Neurologic and psychiatric signs and symptoms in COVID as compared to past pandemics	Long COVID sequelae are similar in regards to chronic fatigue from other viral infections	Chronic fatigue syndrome occurs as a result of viral infections																		
												Reza-Raldivar et al. [10], 2020	Review	N/A	Pathogenicity of COVID and how it affects CNS	COVID affects CNS and it enters through various routes	CNS involvement can lead to chronic signs and symptoms in patients recovered from COVID												
Komaroff et al. [11], 2021	Review	N/A	How post-COVID is similar to myalgic encephalomyelitis	Autoantibody formation against various cell lines can be the cause of post-COVID severity of disease	Long-term effects of COVID on the body may be due to the damage caused to the vital organs or the psychological insult due to the pandemic																								
						Yong et al. [12], 2021	Review	N/A	The sequelae of long-standing COVID infection	Long COVID may be due to dysfunction of T and B cells	Long COVID is a serious problem that needs to be addressed						
																		Akbarialiabad et al. [13], 2021	Systematic review	N/A	Understanding the effect of long COVID	All vital organs are affected due to COVID, still need a proper definition of the long COVID	COVID effects multiple organs of the body resulting in long-term effects
																								Raveendran et al. [14], 2021	Review	N/A	Defining what long COVID is and its long-standing symptoms	Fatigue, respiratory symptoms, loss of concentration, and body aches constitute LONG COVID	How we can tackle this long COVID
Desai et al. [15], 2021	Review	N/A	Study the involvement of different body organs in COVID	More data is required as the virus is changing its strains rapidly	Involvement of various organs occurs in COVID																								
						Yang et al. [16], 2021	Review	N/A	CNS insult caused by COVID	COVID causes a wide variety of signs and symptoms, CNS involvement is the most common	COIVD gains entry by ACE, neural transport, and through hematogenous route, causing multiple signs and symptoms																		
												Lopez-Leon et al. [17], 2021	Meta-analysis	N/A	Effects of COVID in chronic cases	Almost all major organs are affected by COVID	COVID causes a wide variety of long-standing effects on the human body												
																		Systematic review
Dennis et al. [18], 2021	Observational study	201	To study the extent of organ damage in patients who contracted COVID	Multiple organs were affected as a result of COVID	Patients with less severity of disease experience at least one organ dysfunction for at least a couple of months.													
						Bougakov et al. [19], 2021	Review	N/A	How COVID affects the nervous system	Virus uses different mechanisms to enter the brain, thus causing long-standing effects like headache, cognitive impairment, and many more symptoms	COVID affects CNS causing a wide array of signs and symptoms						
												Agergaard et al. [20], 2021	Clinical trial	23	Effect of COVID on the muscles and nerves	The study showed muscle changes post COVID	COVID can affect multiple organs including the muscles, thus leading to fatigue and weakness
																		Banerjee et al. [21], 2020	Review	N/A	Effects of COVID on the brain and psychological health	COVID causes a wide variety of signs and symptoms on the CNS	COVID can present in various ways like headache, loss of taste and smell, causing various psychological signs and symptoms
Yong et al. [22], 2021	Review	N/A	How COVID affects brainstem	Brain stem dysfunction maybe be linked to the long COVID syndrome, still a hypothesis	Brainstem dysfunction could be related to the chronic effects of COVID on the body																		
																								Asadi-Pooya et al. [23], 2021	Observational study	4681	What factors lead to chronic COVID	Study showed that the extent of the disease depends on the sex of the patient, the presence and absence of respiratory symptoms, and the days spent in the hospital	Long-standing COVID is associated with various signs and symptoms and it depends on various factors, the most important being the sex of the patient, the presence of pulmonary symptoms
						Kirwan et al. [24], 2020	Review	N/A	Muscle loss in long-standing COVID	COVID has lead to effects on various organs of the body including muscles, leading to muscle loss	Spending more time indoors leads to an increased buildup of fats and decreased physical activity																		
												Romero-Duarte et al. [25], 2021	Observational study	969	Long-term symptoms and the fate of patients recovered from COVID	Patients recovered from COVID experienced multiple signs and symptoms ranging from mild to severe on the basis of severity	Steps can be taken to prevent the long-term effects in patients who tested positive for COVID so that they can be prevented from the long-standing effects												
Alam et al. [26], 2021	Review	N/A	COVID can lead to effects on the CNS	CNS is being affected by COVID causing multiple signs and symptoms	Work should be done on COVID to find out its effects on the CNS																								
																		Van Herck et al. [27], 2021	Clinical study	239	Persistent fatigue in patients with long COVID	Out of all the patients, females had more signs and symptoms of fatigue post-COVID	Fatigue is the most common long-term manifestation of COVID affecting both physical and the mental health
																								Adeloye et al. [28], 2021	Review	N/A	Effects of long COVID in patients with respiratory diseases both new and old conditions	Patients with underlying respiratory diseases may have more effects of COVID on their body	Underlying respiratory diseases could lead to long-term effects of COVID on the body
Fisicaro et al. [29], 2021	Review	N/A	Involvement of nervous system in COVID	Evidence is still not clear on the basis of Histo-pathological findings from brain tissues as very few subjects showed the evidence	More studies showed be carried out to find the accurate association between COVID and its effect on the tissues on the body																								
						Silva Andrade et al. [30], 2021	Review	N/A	Complications due to long COVID	All major organs are affected by COVID	Thrombosis, neurologic, pulmonary, gastrointestinal, skin, and muscular tissues all are affected by COVID																		

Limitations

As this disease is still new, we have a lot to learn. Most of the studies showed the prevalence of long COVID symptoms in people already with psychiatric disorders, and it is more prevalent in the female population; this creates bias of whether there was any association with the body physique or hormonal changes that contributed to this finding, or if the prevalence of more autoimmune diseases in females lead to these symptoms. More evidence is required to show the association between autoimmune diseases and COVID-19. In order to assess fatigue, there is a need for a standard questionnaire, which should be used to grade fatigue without bias. The role of steroid use in patients with severe COVID-19 needs attention. As corticosteroids can disrupt the cytokine storm, resulting in reduced signs and symptoms, no clear evidence was found that showed whether fatigue still persists in patients who used steroids. We still do not have enough data showing which ethnicity is more affected. We still do not have enough data showing which ethnicity is affected more and the role of antibodies in the severity of acute disease. More information is needed regarding the type of antibody that is more prevalent in causing these long COVID signs and symptoms. Information from primary care providers could be an asset in collecting statistics on long-term signs and symptoms and following up with patients. There is a need for more observational studies, case reports, and case series to answer all these questions thoroughly.

## Conclusions

This virus is still a challenge as it is evolving into different variants with every coming day. Vaccines are out there, and the general population needs to be counseled on the benefits of the vaccine as it can not only prevent the need for hospitalizing but also it can help in preventing patients from going into the long sequelae requiring bed rest and thus causing muscle wasting and severe debilitating fatigue.

The toll on the body post-COVID is both mental and physical. A team of specialists, including physical therapists, nutritionists, primary care providers, nurses, and home care attendants, needs to work hand in hand to provide the best care for this group of people, especially the elderly. They need counseling, physical rehabilitation, regular physical therapy sessions, and of course, nutrient-rich diets to ensure that these groups of people can resume their daily activities of living without depending on someone and can enjoy life to the fullest.
